# Mobile-assisted focus on forms in English for academic purposes instruction: Investigating the impacts on learning academic words

**DOI:** 10.3389/fpsyg.2023.1071555

**Published:** 2023-01-16

**Authors:** Ismail Xodabande, Tahereh Boroughani

**Affiliations:** ^1^Department of Foreign Languages, Kharazmi University, Tehran, Iran; ^2^College of Education and Human Development, Texas A&M University, College Station, TX, United States

**Keywords:** academic vocabulary, intentional vocabulary learning, flashcards, focus on forms instruction, instructed second language acquisition, mobile assisted language learning

## Abstract

Focus on forms (FonFs) is a pedagogical approach in Instructed Second Language Acquisition (ISLA) that emphasizes students’ conscious and direct attention to learning target language features in isolation and outside their meaningful context. FonFs has been employed extensively in foreign language vocabulary instruction, and earlier studies reported positive results for such interventions. The present study investigated mobile-assisted FonFs in the context of English for Academic Purposes (EAP) to address the vocabulary learning needs of Iranian EFL students and examined developments in receptive and productive knowledge of academic words. In doing so, the participants in the experimental learning condition (*N* = 22) were exposed to academic vocabulary using digital flashcards on their mobile phones, and those in the control group (*N* = 15) used word lists. The participants’ vocabulary knowledge was tested using different measures before and after the treatments, and the results were compared using multivariate analysis of variance (MANOVA). The findings indicated that mobile-assisted FonFs was effective in receptive and productive vocabulary learning, and the experimental group outperformed the control group in the post-tests. The effect size of the observed differences was also large; however, differences in productive aspects of academic vocabulary knowledge were associated with smaller learning effects for mobile-assisted FonFs. The study contributes to the growing body of knowledge on mobile-assisted language learning and highlights some implications for teaching academic vocabulary *via* mobile-assisted FonFs.

## Introduction

Instructed second language acquisition (ISLA) is an active research area aiming to understand issues with significant relevance to learning and teaching in instructional environments (Loewen, 2015). Instructed second language acquisition also examines a range of pedagogical interventions designed and implemented to support second/foreign language learners in developing their knowledge and competencies across various skills more effectively (Sato and Loewen, 2019). Among different aspects of the long-term process of literacy development in learning English as a Foreign Language (EFL), addressing vocabulary learning needs has remained a consistent pedagogical concern (Nation, 2013; Webb and Nation, 2017). In this regard, previous research indicated that after years of being exposed to instructional materials and classroom teaching, there are considerable gaps in the lexical knowledge of EFL students, and they mostly struggle in learning a significant proportion of high-frequency words in English, which is essential for their successful communication in the target language (Webb and Chang, 2012; Rahmani et al., 2022; Zakian et al., 2022; Xodabande et al., 2022c). Additionally, EFL students face serious challenges in terms of learning and using academic (or semi-technical) vocabulary, a group of medium-frequency words that are employed more frequently in academic discourse for describing abstract ideas and processes (Coxhead, 2000, 2019; Evans and Morrison, 2010, 2011; Paquot, 2010; Xodabande et al., 2022a). Accordingly, since vocabulary knowledge is the most important factor in language learning, which correlates positively with developments in both receptive and productive uses of language (Clenton and Booth, 2020), ISLA has a particular interest in designing effective interventions for scaffolding vocabulary knowledge development among language learners (González-Fernández and Schmitt, 2017). Consequently, this study aimed to investigate the effectiveness of a mobile-assisted intervention for learning academic vocabulary by university students. The research contributes to the growing body of knowledge on mobile-assisted vocabulary learning and sheds lights on the effectiveness of this approach in scaffolding EFL learners’ academic literacy development.

## Literature review

### Theoretical background

Second language vocabulary development takes place through two learning mechanisms that involve incidental and intentional learning (Webb and Nation, 2017; Nation, 2022; VanPatten and Smith, 2022). In this regard, incidental vocabulary learning happens as a by-product of meaningful interactions which expose language learners to a large amount of comprehensible input. More specifically, in this mechanism, the focus of language use (and interaction) is on communicating meaning without paying explicit attention to language usage. Consequently, incidental vocabulary learning is a long-term process and needs large amounts of input. In contrast, intentional learning is associated with conscious and direct attention to language forms. Considering the fact that in most EFL learning contexts, providing language learners with the huge amount of input required for incidental learning is not easily possible, researchers argued that for helping students in developing adequate vocabulary for successful communication in the target language, there is a need for prioritizing intentional vocabulary learning in instructional programs (Vilkaitė-Lozdienė and Schmitt, 2019). There are a number of resources for scaffolding intentional vocabulary learning, and some of the widely used resources include course books and classroom materials, word lists, flashcards, various vocabulary learning activities, and educational games.

Mobile-assisted learning is an approach to intentional vocabulary learning that provide EFL learners with various affordances to augment their L2 literacy developments more effectively. Here, it has been argued that learning words with mobile devices is an inherently motivating activity that impacts the learning outcomes (Stockwell, 2013; Rahmani et al., 2022; Zakian et al., 2022). Considering the crucial role of motivation in language learning (Ushioda and Dörnyei, 2012), this affordance of mobile-assisted learning is of significant importance in scaffolding EFL learners’ vocabulary development. Moreover, mobile-assisted learning might be regarded as a pedagogical intervention to deliver vocabulary learning tasks with high level of involvement. Previous studies indicated that such involvement results in improved vocabulary learning outcomes and also more effective retention of the learned vocabulary items (Laufer and Hulstijn, 2001; Yanagisawa and Webb, 2021; Lei and Reynolds, 2022; Liu and Reynolds, 2022). Additionally, repeated encounters with the target vocabulary items is a crucial factor in L2 vocabulary learning (Webb and Nation, 2017), and mobile-assisted learning environments provide learners with some affordances to recycle the learned items and increase the level of uptake from input. Accordingly, using mobile devices for intentional vocabulary learning is a promising approach to augment EFL students’ language learning.

### Focus on forms (FonFs) in vocabulary instruction

Form-focused instruction (FFI) is an approach within ISLA that “draws learners’ attention to language form during communicative activities either implicitly or explicitly when their primary focus is to communicate meaning” (Sato and Loewen, 2019, p. 5). Although the concept originated in the context of teaching grammar and developed out of a general dissatisfaction with learning outcomes in terms of grammatical competence in communicative language teaching (Laufer and Girsai, 2008), this pedagogical approach has been applied to vocabulary instruction too (Hill and Laufer, 2003; Laufer, 2005, 2006). Accordingly, studies investigated the impacts of directing learners’ attention to single words and multi-word vocabulary items within communicative tasks (De la Fuente, 2002) or investigated learning words in isolation and outside their meaningful contexts (Webb, 2007). The latter approach has been referred to as Focus on Forms (FonFs) in vocabulary instruction (Laufer and Girsai, 2008), and the existing literature strongly supports the beneficial nature of both approaches in developing vocabulary knowledge of language learners (Laufer, 2005; Nation, 2013; Nakata, 2019). In recent years, significant developments in computer- and mobile-assisted language learning expanded the repertoire of available tools and options for tailoring FFI on vocabulary development (Mahdi, 2017; Lin and Lin, 2019; Hao et al., 2021).

Moreover, with the global expansion and dominance of English as the academic *lingua franca* (Hyland, 2009), there is a growing interest in developing EFL students’ academic skills that are essential for their research publication needs (Flowerdew, 2015, 2019; Li and Flowerdew, 2020). Accordingly, as academic vocabulary covers a significant proportion (i.e., around 10%) of target texts that university students need to read and write (Coxhead, 2011, 2019), mobile-assisted FonFs might be considered a practical approach for teaching academic words. Nevertheless, the scope of research in this area remained largely limited, and the affordances of mobile devices for teaching academic vocabulary are relatively underutilized (Dizon, 2016; Ashcroft et al., 2018; Xodabande and Atai, 2022). Additionally, among the various strategies and activities developed for FonFs in vocabulary instruction, word cards (or flashcards) attracted considerable attention (Webb and Nation, 2017; Nakata, 2019; Lei and Reynolds, 2022). This strategy provides learners with an effective and efficient way to learn a large number of words over a short time (Nation, 2013), and empirical evidence suggests that flashcard-based learning is more effective compared to learning words from word lists or even from context (Webb and Nation, 2017; Webb et al., 2020). Additionally, this strategy contributes significantly to developments in both receptive and productive learning of vocabulary items (Li and Hafner, 2022) which is of significant importance in EAP (Coxhead, 2019).

Over the past years, several studies explored learning outcomes from mobile-assisted FonFs on EFL students’ academic vocabulary development (Dizon, 2016; Ashcroft et al., 2018; Xodabande and Atai, 2022; Xodabande et al., 2022b). For example, Dizon (2016) explored the learning gains from using an online tool for learning academic vocabulary among Japanese university students. The study findings indicated that the participants considered mobile applications easy to use and significantly improved their vocabulary knowledge. Additionally, Ashcroft et al. (2018) studied the effectiveness of the FonFs approach in teaching academic words using digital and paper flashcards among EFL students at different English proficiency levels. The results revealed that both interventions were effective for high-proficient students; nevertheless, low-proficient students benefited more from using digital flashcards. In another study, Xodabande and Atai (2022) investigated the learning outcomes of using digital flashcards with spaced repetition technology for teaching academic words to EFL university students. In line with earlier findings, this study also reported significant vocabulary gains for FonFs using mobile devices and digital flashcards. Recently, Xodabande et al. (2022b) compared three FonFs interventions for teaching academic words among Iranian university students. Accordingly, they explored learning outcomes from digital flashcards on mobile devices, paper-based cards, and word lists. The findings of the study provided further empirical evidence for the effectiveness of mobile-assisted FonFs in academic vocabulary development.

### The present study

Despite increased interest in FonFs in mobile-assisted vocabulary instruction, there are some gaps in this growing body of knowledge that demand further empirical research. First, although FonFs is a promising approach for teaching a large number of words, mobile-assisted interventions were mostly conducted in short-time periods for teaching a small number of words (Lin and Lin, 2019). Second, in terms of research design, the lack of control groups in some interventions limited the generalizability of the findings beyond the context of the studies (Lin and Lin, 2019). Third, despite the multifaceted nature of vocabulary knowledge, most studies were concerned with improvements in receptive knowledge of the target words (i.e., developments in vocabulary size), and the affordance of mobile assisted FonFs for productive vocabulary knowledge remained far less explored (Li and Hafner, 2022). The present study aimed to address these gaps and investigated mobile-assisted FonFs intervention in EAP instruction. More specifically, the study compared learning outcomes from using digital flashcards and word lists for developing both receptive and productive knowledge of academic vocabulary by addressing the following research questions:

1. Does mobile-assisted FonFs result in significant improvements in university students’ academic vocabulary?
2. Does mobile-assisted FonFs result in significant improvements in productive knowledge of academic words?

## Method

### Participants

The study participants were 37 adult English as a Foreign Language (EFL) learners in a private language teaching institute in Tehran, Iran. The mean age of the participants was 21, and they were selected based on the convenience sampling procedure and their availability in the study context. The general proficiency level of the participants in English was assessed using the reading and listening sections of a sample IELTS test, and the results showed that most of them were at intermediate level based on the Common European Framework of Reference for Languages (CEFR; Council of Europe, 2001). Following Li and Hafner (2022), the participants were assigned to experimental and control conditions based on their own preferences for using different materials. Accordingly, 22 students preferred learning academic words on their smartphone with digital flashcards (experimental group), and 15 participants opted for using traditional materials (paper-based vocabulary lists). Informed consent for participating in the study were obtained from the students before the treatment.

### Materials and testing instruments

The Academic Word List (AWL; Coxhead, 2000) was used as the English academic vocabulary source. The list is widely employed in instructional materials development and testing in English for Academic Purposes (EAP) programs (Coxhead, 2011; Schmitt and Schmitt, 2011; McLean and Kramer, 2015). The AWL has 570 word families and around 3,000 individual words. The current study focused on 480 words appearing in the first book from a three-volume series dedicated to learning the AWL (Coxhead and Nation, 2018). These words are the most frequently used academic vocabulary in English, and learning them can contribute significantly to the comprehension of academic discourse.

Two types of learning materials were used in the study. The participants in the experimental group were given ready-made digital flashcards in 16 sets (representing different units in the textbook). These participants used Anki which is free and open-source learning application for android mobile devices (AnkiDroid, 2020). More specifically, Anki is a digital flashcard app with a built-in spaced repetition system that supports more effective and long-term vocabulary learning. Learners are able to create their own flashcards or use ready-made sets (for more information see the following website: https://www.tofugu.com/reviews/anki/). Each digital flashcard developed using Anki app for the current study contained the target academic word, Persian translation, the definition in English, and two sample sentences featuring the words used in a meaningful context. Using Anki helped the participants to study these academic words in a mobile-assisted learning environment. The participants in the control group were given 16 paper-based word lists containing the same information as provided in digital flashcards. Accordingly, the participants were exposed to the same content; nevertheless, the learning environment was different for the experimental and control learning conditions.

In order to assess changes before and after the treatment, a number of testing instruments were used to test both receptive and productive dimensions of academic vocabulary knowledge among the participants (Milton, 2009). First, to create representative and balanced tests for measuring the changes in the participants’ vocabulary knowledge, the 480 target words were randomly assigned into three sets with 160 items. Second, since testing all items in the three sets was not practical, 80 words were selected randomly from these sets for designing six short tests (three pre-tests and three post-tests). Accordingly, following Wu (2015), two multiple-choice item vocabulary tests were designed to test receptive vocabulary knowledge, each containing 40 items (Cronbach’s alpha = 0.79). The second test measured productive knowledge of academic words by giving the students definitions and translations of the words and asking them to provide the appropriate academic word. The third test required the participants to identify the context of use by filling in blanks in sentences with academic words (Cronbach’s alpha = 0.84). To ensure the validity of the instruments, the following steps were taken. First, the development of the test items was based on random selection of the words to increase the representativeness of the tests with respect to the target items (i.e., 480 words from the AWL). Second, the tests and associated items were developed using guidelines provided in scholarly publications for testing different aspects of vocabulary knowledge (Milton, 2009). Third, the test items were reviewed by two experts with extensive experience in language testing and their feedback resulted in revising or rewriting some items. Finally, the three instruments were piloted to a sample group of EFL students (*N* = 20) to analyze the items for their difficulty and discrimination.

### Procedures and data analysis

The study was carried out over 3 months, and data collection started by administrating the pre-tests. During this period, both groups received classroom instruction based on the curriculum implemented by the institute for preparing the students for the international exams (i.e., IELTS and TOEFL). The classes were held twice weekly, and each session lasted for 70 min. In addition to covering the regular syllabus, 20 min of classroom time in all sessions focused on teaching academic words (Coxhead and Nation, 2018). Additionally, the participants were asked to review target academic words covered in the classroom (60 words every week) using their preferred materials (i.e., digital flashcards and paper-based word lists) outside the classroom. The participants were informed that 30% of their overall evaluation would be based on their scores on post-treatment vocabulary tests. Data collection ended with measuring and documenting changes in the participants’ vocabulary knowledge in the post-tests.

Data analysis was performed using IBM SPSS statistics version 25. In doing so, both descriptive and inferential statistical techniques were used. For descriptive statistics, mean values, standard deviations, and standard error of mean were obtained for the data. For inferential statistics, the scores on vocabulary tests were analyzed using multivariate analysis of variance (MANOVA; Pallant, 2016). MANOVA is an extension of variance analysis used when there is more than one dependent variable, and these variables are related to each other either conceptually or in a specific way. Accordingly, since the current study used three tests for measuring different aspects of academic vocabulary knowledge, the scores obtained by the experimental and control groups were compared using MANOVA.

## Results

The results of descriptive statistics are summarized in Table 1. As it is represented below in the mean values for the pre-tests, the experimental and control groups obtained similar scores on three measures namely receptive, productive, and filling in blanks (context) tests. The only notable difference was in the scores in the context test, as the control group (*M* = 17.47, SD = 2.26) scored higher than the experimental group (*M* = 16.41, SD = 2.88). However, the results obtained on the post-tests pointed to a different pattern. Accordingly, although both groups improved their scores considerably compared to the pre-tests, the experimental group participants obtained higher scores than the control group.

**Table 1:** Descriptive statistics for the scores obtained on pre- and post-tests.

Group statistics						
		Group	*N*	Mean	Std. deviation	Std. error mean
Pre-tests	Receptive	Experimental	22	13.68	2.982	0.636
		Control	15	13.27	2.463	0.636
	Productive	Experimental	22	6.18	2.922	0.623
		Control	15	6.67	3.016	0.779
	Context	Experimental	22	16.41	2.889	0.616
		Control	15	17.47	2.264	0.584
Post-tests	Receptive	Experimental	22	26.55	2.790	0.595
		Control	15	23.67	3.063	0.791
	Productive	Experimental	22	10.82	3.554	0.758
		Control	15	8.13	2.722	0.703
	Context	Experimental	22	28.09	5.588	1.191
		Control	15	24.33	3.994	1.031

The results of multivariate tests comparing the pre-test scores pointed to no significant difference in the two groups’ performances on three tests in general, Wilks’ Lambda = 0.953, *F* (3, 33) = 0.548, *p* = 0.653, multivariate *η_p_*^2^ = 0.047. Additionally, the results obtained for between-subjects effects indicated that the observed differences in scores obtained on each measure were not statistically significant (Table 2).

**Table 2:** Tests of between-subjects effects for the scores on pre-tests.

Source	Dependent variable	Type III sum of squares	*df*	Mean square	*F*	Sig.	Partial eta squared
Group	Pre-test (receptive)	1.537	1	1.537	0.198	0.659	0.006
	Pre-test (productive)	2.097	1	2.097	0.239	0.628	0.007
	Pre-test (context)	9.976	1	9.976	1.413	0.243	0.039

The results for multivariate tests comparing the experimental and control groups on the post-tests are shown in Table 3. In this regard, the findings indicated that considering the three measures together, the observed differences in the scores were statistically significant, Wilks’ Lambda = 0.659, *F* (3, 33) = 5.697, *p* ≤ 0.001, multivariate *η_p_*^2^ = 0.341. The effect size of the differences was also very large based on criteria proposed by Cohen (1988).

**Table 3:** Multivariate tests^a^ for the scores obtained on post-tests.

Effect		Value	*F*	Hypothesis *df*	Error *df*	Sig.	Partial eta squared
Intercept	Pillai’s Trace	0.990	1087.642^b^	3.000	33.000	0.000	0.990
	Wilks’ Lambda	0.010	1087.642^b^	3.000	33.000	0.000	0.990
	Hotelling’s Trace	98.877	1087.642^b^	3.000	33.000	0.000	0.990
	Roy’s Largest Root	98.877	1087.642^b^	3.000	33.000	0.000	0.990
Group	Pillai’s Trace	0.341	5.697^b^	3.000	33.000	0.003	0.341
	Wilks’ Lambda	0.659	5.697^b^	3.000	33.000	0.003	0.341
	Hotelling’s Trace	0.518	5.697^b^	3.000	33.000	0.003	0.341
	Roy’s Largest Root	0.518	5.697^b^	3.000	33.000	0.003	0.341

a Design: intercept + group.

b Exact statistic.

Finally, the results for between-subjects effects on the post-tests revealed that the differences observed on receptive (*F* (1, 35) = 8.776, *p* ≤ 0.005, *η_p_*^2^ = 0.2), productive (*F* (1, 35) = 6.098, *p* ≤ 0.019, *η_p_*^2^ = 0.148), and fill in the blank (context; *F* (1, 35) = 5.013, *p* ≤ 0.032, *η_p_*^2^ = 0.125) tests were statistically significant (Table 4).

**Table 4:** Tests of between-subjects effects for the scores obtained on post-tests.

Source	Dependent variable	Type III sum of squares	*df*	Mean square	*F*	Sig.	Partial eta squared
Group	Post-test (receptive)	73.915	1	73.915	8.776	0.005	0.200
	Post-test (productive)	64.291	1	64.291	6.098	0.019	0.148
	Post-test (context)	125.930	1	125.930	5.013	0.032	0.125

## Discussion and conclusion

The present study investigated the impacts of FonFs in EAP instruction for developing the knowledge of academic words among EFL learners. The first research question was concerned with the effectiveness of FonFs in improving university students’ academic vocabulary. Accordingly, learning outcomes from two interventions, namely mobile-assisted FonFs *via* digital flashcards and FonFs using word lists, were compared. Data analysis revealed that before the treatment, participants in the experimental and control learning conditions were similar with respect to their knowledge of academic words. Nevertheless, the results obtained on the post-tests showed that the participants receiving mobile-assisted FonFs instruction outperformed those in the control group (FonFs using word lists) and learned more words. These findings align with earlier studies that reported positive learning outcomes for the effectiveness of mobile-assisted FonFs in scaffolding academic vocabulary development (Dizon, 2016; Ashcroft et al., 2018; Xodabande and Atai, 2022; Xodabande et al., 2022b). More specifically, similar to the findings reported by Xodabande and Atai (2022), this study also indicated that students who received mobile-assisted FonFs improved their vocabulary knowledge significantly. Moreover, in line with Xodabande et al. (2022b) the results of the present study pointed to considerable learning outcomes for using digital flashcards in comparison with traditional materials. Although Dizon (2016) explored the learning outcomes from using online digital flashcards, the findings of the current study confirmed that both online and offline approaches yield to significant learning outcomes. Finally, with respect to the findings reported by Ashcroft et al. (2018) that highlighted the effectiveness of mobile-assisted FonFs for low-proficient EFL learners, this study reports empirical evidence for the effectiveness of this strategy for intermediate EFL students.

The improvements in the participants’ vocabulary knowledge observed in this study might have resulted from a number of factors. First, concerning the learning mechanism, both groups employed intentional learning strategies to learn the target academic words, and as previous research indicated, such strategies are more effective than incidental learning mechanisms that require large amounts of input (Webb and Nation, 2017). Hence, the findings of the current study provided further empirical support for the effectiveness of intentional learning approach in mobile-assisted FonFs. Second, considering the motivational affordance of the mobile-assisted learning, learning with digital flashcards enhanced the participants’ motivation which might has resulted in significant learning outcomes. As the participants were studying to take the IELTS exam in the near future, learning academic vocabulary was also among their language learning needs. Hence, both groups were highly motivated to learn target vocabulary items. In this regard, it seems that mobile-assisted FonFs might be specifically beneficial for those EAP students who need to improve their academic vocabulary in a short time period for international examinations. Third, learning academic vocabulary was integrated into the syllabi of the EAP course offered for the participants, and learning vocabulary items inside the classroom and then reviewing those words in self-regulated learning outside the classroom increased the effectiveness of the instructional approach. This affordance of mobile-assisted learning facilitated and promoted the level of the students’ task involvement that is essential for long-term vocabulary development (Liu and Reynolds, 2022). Consequently, considering the importance of academic vocabulary for university students and also significant learning gains observed for mobile-assisted FonFs, it seems that this pedagogical approach can compensate for inadequate coverage of academic vocabulary in EAP programs and facilitate students’ academic literacy development by using the affordances of mobile-assisted learning for extending the vocabulary learning beyond the classroom.

The second research question examined the contribution of mobile-assisted FonFs on receptive and productive vocabulary development. In this regard, data analysis revealed that mobile-assisted FonFs improved both receptive and productive knowledge of academic words, as the experimental group outperformed the control group in all three measures (Tables 3, 4). Accordingly, the findings of the current study provide further empirical evidence for the effectiveness of mobile-assisted FonFs in developing productive vocabulary knowledge (Li and Hafner, 2022; Xodabande and Atai, 2022). However, data analysis (Table 4) also indicated that the magnitude of the observed differences (i.e., effect size) was disproportionate for receptive and productive learning gains. In this regard, although the effect size of the differences between mobile-assisted FonFs and learning from word lists was very large for receptive knowledge of academic words (*η_p_*^2^ = 0.20), the associated effect size for productive knowledge was 0.148, which is considerably smaller. Furthermore, the effect size of the differences in the third measure that required participants to identify the appropriate context of use for the academic words was even smaller (i.e., 0.125).

Consequently, the findings of the current study show that although mobile-assisted FonFs resulted in significant development in receptive and productive vocabulary knowledge, the strategy was more effective for the former. Such findings might have resulted from the following reasons. First, given that flashcards and word lists are strategies primarily developed for learning form-meaning connections, the relative advantage of the interventions for receptive knowledge development is inevitable. Second, the participants in the experimental group used digital flashcards that provided them with some additional affordances for vocabulary learning including the spaced repetition system. This affordance of mobile-assisted learning provided them with a systematic approach to recycling academic words, and consequently facilitated more meaningful encounters with the target words. Accordingly, increased encounters resulted in developments in both receptive and productive knowledge of academic words (Nakata, 2019; Lei and Reynolds, 2022). In light of these considerations, it seems that although digital flashcards have significant potential to augment academic vocabulary knowledge, language teachers and university students need to be aware of the various affordances provided by such platforms, and consequently aim to tailor those affordances to learning needs. As developing the productive aspect of academic vocabulary needs more practice and active retrieval, DFs might be optimized for facilitate this process (i.e., by giving the meaning for recalling the target words).

The study has some implications for teaching academic words in FonFs instruction. English for Academic Purposes (EAP) has been defined as “specialized English-language teaching grounded in the social, cognitive, and linguistic demands of academic target situations, providing focused instruction informed by an understanding of texts and the constraints of academic contexts” (Hyland, 2006, p. 2). Accordingly, given the significant role of academic vocabulary in texts and target situations in using English as the academic *lingua franca*, mobile-assisted FonFs should be considered an effective strategy for augmenting and scaffolding academic literacy development. Given that this instructional approach improves productive knowledge of academic words, there is a need to consider it in EAP materials development and practice. Second, mobile-assisted FonFs might be implemented as a complementary intervention for classroom EAP instruction. In this case, the affordances of mobile devices for extending learning to anytime and anyplace provide EAP students with practical strategies for addressing their academic vocabulary learning needs (Xodabande and Atai, 2022). Additionally, since integrating new technologies in language teaching is inherently motivating (Stockwell, 2013), mobile-assisted FonFs provide EAP teachers with new ways to increase and sustain students’ motivation for vocabulary learning.

The study had some limitations that should be acknowledged too. First, the sample size investigated in the study was small, and considering the convenience sampling procedure employed, the generalizability of the findings might be limited. Moreover, the study used a pre-test and post-test design, and further research is required to investigate the delayed impacts of mobile-assisted FonFs. Finally, the study was mainly concerned with developments in vocabulary knowledge *via* quantitative data, and the participants’ perceptions and attitudes regarding mobile-assisted FonFs remained unexplored. In this regard, the findings of the current study need to be investigated in future investigations. More specifically, there is a need for more research on the affordances of mobile-devices for scaffolding academic vocabulary learning in the long-term. Relatedly, we need more research on the contribution of digital flashcards on productive vocabulary learning as this a major area of concern for most EFL university students. This line of research also needs incorporating mixed-methods approaches to better understand different mechanisms involved in using mobile devices for vocabulary learning. We encourage other researchers to follow up these lines as such explorations shed more light on learning outcomes in mobile-assisted vocabulary learning and results in informed pedagogical practices for FonFs for learning academic words.

## Data availability statement

The original contributions presented in the study are included in the article/supplementary material, further inquiries can be directed to the corresponding author.

## Ethics statement

Ethical review and approval was not required for the study on human participants in accordance with the local legislation and institutional requirements. The patients/participants provided their written informed consent to participate in this study.

## Author contributions

All authors listed have made a substantial, direct, and intellectual contribution to the work and approved it for publication.

## Conflict of interest

The authors declare that the research was conducted in the absence of any commercial or financial relationships that could be construed as a potential conflict of interest.

## Publisher’s note

All claims expressed in this article are solely those of the authors and do not necessarily represent those of their affiliated organizations, or those of the publisher, the editors and the reviewers. Any product that may be evaluated in this article, or claim that may be made by its manufacturer, is not guaranteed or endorsed by the publisher.

## References

[ref1] AnkiDroid. (2020). *AnkiDroid flashcards*. https://play.google.com/store/apps/details?id=com.ichi2.anki&hl=en (Accessed January 5, 2023).

[ref2] Ashcroft R. J. Cvitkovic R. Praver M. (2018). Digital flashcard L2 vocabulary learning out-performs traditional flashcards at lower proficiency levels: a mixed-methods study of 139 Japanese university students. EuroCALL Rev. 26:14. doi: 10.4995/eurocall.2018.7881

[ref3] Clenton J. Booth P. (Eds.). (2020). Vocabulary and the Four Skills: Pedagogy, Practice, and Implications for Teaching Vocabulary. London: Routledge.

[ref4] Cohen J. (1988). Statistical Power Analysis for the Behavioral Sciences. London: Routledge.

[ref5] Council of Europe. (2001). Common European framework of reference for languages: Learning, Teaching, Assessment. Cambridge: Cambridge University Press.

[ref6] Coxhead A. (2000). A new academic word list. TESOL Q. 34, 213–238. doi: 10.2307/3587951

[ref7] Coxhead A. (2011). The academic word list 10 years on: research and teaching implications. TESOL Q. 45, 355–362. doi: 10.5054/tq.2011.254528

[ref8] Coxhead A. (2019). “Academic vocabulary” in The Routledge Handbook of Vocabulary Studies. ed. Webb S. (London: Routledge), 97–110.

[ref9] Coxhead A. Nation I. S. P. (2018). Reading for the Academic World 1. Seongnam: Seed Learning.

[ref10] De la Fuente M. J. (2002). Negotiation and oral acquisition of L2 vocabulary: the roles of input and output in the receptive and productive acquisition of words. Stud. Second. Lang. Acquis. 24, 81–112. doi: 10.1017/S0272263102001043

[ref11] Dizon G. (2016). Quizlet in the EFL classroom: enhancing academic vocabulary acquisition of Japanese university students. Teach. Eng. Technol. 16, 40–56.

[ref12] Evans S. Morrison B. (2010). The first term at university: implications for EAP. ELT J. 65, 387–397. doi: 10.1093/elt/ccq072

[ref13] Evans S. Morrison B. (2011). Meeting the challenges of English-medium higher education: the first-year experience in Hong Kong. Engl. Specif. Purp. 30, 198–208. doi: 10.1016/j.esp.2011.01.001

[ref14] Flowerdew J. (2015). Some thoughts on English for research publication purposes (ERPP) and related issues. Lang. Teach. 48, 250–262. doi: 10.1017/S0261444812000523

[ref15] Flowerdew J. (2019). The linguistic disadvantage of scholars who write in English as an additional language: myth or reality. Lang. Teach. 52, 249–260. doi: 10.1017/S0261444819000041

[ref16] González-Fernández B. Schmitt N. (2017). “Vocabulary acquisition” in The Routledge Handbook of Instructed Second Language Acquisition. eds. Loewen S. Sato M. (London: Routledge), 280–298.

[ref17] Hao T. Wang Z. Ardasheva Y. (2021). Technology-assisted vocabulary learning for EFL learners: a meta-analysis. J. Res. Educ. Effect 14, 645–667. doi: 10.1080/19345747.2021.1917028

[ref18] Hill M. M. Laufer B. (2003). Type of task, time-on-task and electronic dictionaries in incidental vocabulary acquisition. Int. Rev. Appl. Linguist. 41, 87–106. doi: 10.1515/iral.2003.007

[ref19] Hyland K. (2006). English for Academic Purposes: An Advanced Resource Book. London: Routledge.

[ref20] Hyland K. (2009). Academic Discourse: English in a Global Context. London: Bloomsbury.

[ref21] Laufer B. (2005). “Focus on form in second language vocabulary learning” in EUROSLA Yearbook. eds. Foster-Cohen S. H. Mayo D. P. G. Cenoz J., vol. 5 (Amsterdam: John Benjamins Publishing Company), 223–250.

[ref22] Laufer B. (2006). Comparing focus on form and focus on form S in second language vocabulary learning. Can. Mod. Lang. Rev. 63, 149–166. doi: 10.3138/cmlr.63.1.149

[ref23] Laufer B. Girsai N. (2008). Form-focused instruction in second language vocabulary learning: a case for contrastive analysis and translation. Appl. Linguist. 29, 694–716. doi: 10.1093/applin/amn018

[ref24] Laufer B. Hulstijn J. (2001). Incidental vocabulary acquisition in a second language: the construct of task-induced involvement. Appl. Linguist. 22, 1–26. doi: 10.1093/applin/22.1.1

[ref25] Lei Y. Reynolds B. L. (2022). Learning English vocabulary from word cards: a research synthesis. Front. Psychol. 13, 1–26. doi: 10.3389/fpsyg.2022.984211, PMID: 36148102 PMC9485613

[ref26] Li Y. Flowerdew J. (2020). Teaching English for research publication purposes (ERPP): a review of language teachers’ pedagogical initiatives. Engl. Specif. Purp. 59, 29–41. doi: 10.1016/j.esp.2020.03.002

[ref27] Li Y. Hafner C. A. (2022). Mobile-assisted vocabulary learning: investigating receptive and productive vocabulary knowledge of Chinese EFL learners. ReCALL 34, 66–80. doi: 10.1017/S0958344021000161

[ref28] Lin J.-J. Lin H. (2019). Mobile-assisted ESL/EFL vocabulary learning: a systematic review and meta-analysis. Comput. Assist. Lang. Learn. 32, 878–919. doi: 10.1080/09588221.2018.1541359

[ref29] Liu S. Reynolds B. L. (2022). Empirical support for the involvement load hypothesis (ILH): a systematic review. Behav. Sci. 12, 1–23. doi: 10.3390/bs12100354, PMID: 36285923 PMC9598591

[ref30] Loewen S. (2015). Introduction to Instructed Second Language Acquisition. London: Routledge.

[ref31] Mahdi H. S. (2017). Effectiveness of Mobile devices on vocabulary learning: a meta-analysis. J. Educ. Comput. Res. 56, 134–154. doi: 10.1177/0735633117698826

[ref32] McLean S. Kramer B. (2015). The creation of a new vocabulary levels test. Shiken 19, 1–11.

[ref33] Milton J. (2009). Measuring Second Language Vocabulary Acquisition. Clevedon: Multilingual Matters.

[ref34] Nakata T. (2019). “Learning words with flash cards and word cards” in The Routledge Handbook of Vocabulary Studies. ed. Webb S. (London: Routledge), 304–319.

[ref35] Nation I. S. P. (2013). Learning Vocabulary in Another Language (2nd). Cambridge: Cambridge University Press.

[ref36] Nation I. S. P. (2022). Learning Vocabulary in Another Language (3rd). Cambridge: Cambridge University Press.

[ref37] Pallant J. (2016). SPSS Survival Manual: A Step by Step Guide to Data Analysis Using IBM SPSS (6th). London: Routledge.

[ref38] Paquot M. (2010). Academic Vocabulary in Learner Writing: From Extraction to Analysis. London: Continuum International Publishing Group.

[ref39] Rahmani A. Asadi V. Xodabande I. (2022). Using mobile devices for vocabulary learning outside the classroom: improving the English as foreign language learners’ knowledge of high-frequency words. Front. Psychol. 13, 1–7. doi: 10.3389/fpsyg.2022.899885, PMID: 35814053 PMC9263603

[ref40] Sato M. Loewen S. (Eds.) (2019). Evidence-based Second Language Pedagogy. London: Routledge.

[ref41] Schmitt D. Schmitt N. (2011). Focus on Vocabulary 2: Mastering the Academic Word List. London: Pearson Education.

[ref42] Stockwell G. (2013). “Technology and motivation in English-language teaching and learning” in International Perspectives on Motivation. ed. Ushioda E. (London: Palgrave Macmillan), 156–175.

[ref43] Ushioda E. Dörnyei Z. (2012). “Motivation” in The Routledge Handbook of Second Language Acquisition. eds. Gass S. M. Mackey A. (London: Routledge), 396–409.

[ref44] VanPatten B. Smith M. (2022). Explicit and Implicit Learning in Second Language Acquisition. Cambridge: Cambridge University Press.

[ref45] Vilkaitė-Lozdienė L. Schmitt N. (2019). “Frequency as a guide for vocabulary usefulness: high-, mid-, and low-frequency words” in The Routledge Handbook of Vocabulary Studies. ed. Webb S. (London: Routledge), 81–96.

[ref46] Webb S. (2007). Learning word pairs and glossed sentences: the effects of a single context on vocabulary knowledge. Lang. Teach. Res. 11, 63–81. doi: 10.1177/1362168806072463

[ref47] Webb S. Chang A. C.-S. (2012). Second language vocabulary growth. RELC J. 43, 113–126. doi: 10.1177/0033688212439367

[ref48] Webb S. Nation I. S. P. (2017). How Vocabulary is Learned. Oxford: Oxford University Press.

[ref49] Webb S. Yanagisawa A. Uchihara T. (2020). How effective are intentional vocabulary-learning activities? A meta-analysis. Mod. Lang. J. 104, 715–738. doi: 10.1111/modl.12671

[ref310] Wu Q. (2015). Designing a smartphone app to teach English (L2) vocabulary. Comput. Educ. 85, 170–179. doi: 10.1016/j.compedu.2015.02.013

[ref50] Xodabande I. Asadi V. Valizadeh M. (2022a). Teaching vocabulary items in corpus-based wordlists to university students: comparing the effectiveness of digital and paper-based flashcards. J. China Comput.-Assist. Lang. Learn. 1–24. doi: 10.1515/jccall-2022-0016

[ref51] Xodabande I. Atai M. R. (2022). Using mobile applications for self-directed learning of academic vocabulary among university students. Open Learn. J. Open, Dist. e-Learning 37, 330–347. doi: 10.1080/02680513.2020.1847061

[ref52] Xodabande I. Iravi Y. Mansouri B. Matinparsa H. (2022b). Teaching academic words with digital flashcards: investigating the effectiveness of Mobile-assisted vocabulary learning for university students. Front. Psychol. 13, 1–11. doi: 10.3389/fpsyg.2022.893821, PMID: 35774936 PMC9239377

[ref53] Xodabande I. Pourhassan A. Valizadeh M. (2022c). Self-directed learning of core vocabulary in English by EFL learners: comparing the outcomes from paper and mobile application flashcards. J. Comput. Educ. 9, 93–111. doi: 10.1007/S40692-021-00197-6

[ref54] Yanagisawa A. Webb S. (2021). Involvement load hypothesis plus: creating an improved predictive model of incidental vocabulary learning. Stud. Second. Lang. Acquis. 44, 1279–1308. doi: 10.1017/S0272263121000577

[ref55] Zakian M. Xodabande I. Valizadeh M. Yousefvand M. (2022). Out-of-the-classroom learning of English vocabulary by EFL learners: investigating the effectiveness of mobile assisted learning with digital flashcards. Asian-Pac. J. Second Foreign Lang. Educ. 7, 1–16. doi: 10.1186/s40862-022-00143-8

